# Using Benford’s law to investigate Natural Hazard dataset homogeneity

**DOI:** 10.1038/srep12046

**Published:** 2015-07-09

**Authors:** Renaud Joannes-Boyau, Thomas Bodin, Anja Scheffers, Malcolm Sambridge, Simon Matthias May

**Affiliations:** 1 Southern Cross GeoScience, Southern Cross University, Lismore, NSW, 2480, Australia; 2 Earth and Planetary Science, University of California Berkeley, CA, USA; 3 Research School of Earth Sciences, Australian National University, Canberra, ACT, 0200, Australia; 4 Institute of Geography, University of Cologne, 50923, Cologne, Germany; 5 Laboratoire de Géologie de Lyon, Ecole Normale Supérieure de Lyon, Université de Lyon-1, CNRS, 69364 Lyon Cedex 07, France

## Abstract

Working with a large temporal dataset spanning several decades often represents a challenging task, especially when the record is heterogeneous and incomplete. The use of statistical laws could potentially overcome these problems. Here we apply Benford’s Law (also called the “First-Digit Law”) to the traveled distances of tropical cyclones since 1842. The record of tropical cyclones has been extensively impacted by improvements in detection capabilities over the past decades. We have found that, while the first-digit distribution for the entire record follows Benford’s Law prediction, specific changes such as satellite detection have had serious impacts on the dataset. The *least-square misfit measure* is used as a proxy to observe temporal variations, allowing us to assess data quality and homogeneity over the entire record, and at the same time over specific periods. Such information is crucial when running climatic models and Benford’s Law could potentially be used to overcome and correct for data heterogeneity and/or to select the most appropriate part of the record for detailed studies.

Benford’s Law (BL) is an empirically discovered property related to the frequency of first digits (*sensu stricto* numerals from 1 to 9 forming numbers and values) occurring in “real-world” datasets^1^. It states that in certain datasets the leading digit is distributed in a predictable but non-uniform manner. That is, observations with a lower first digit (1, 2, …) occur more often than those with a higher first digit (… 8, 9). This property arises in many situations but is known to occur when the underlying measurements have a log-uniform distribution:
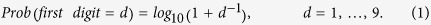


Such datasets are often associated with a power-law distribution with a “heavy tail,” making extreme events far more likely than they would be, for example, in a Gaussian distribution.

Since the initial discovery more than 100 years ago by Newcomb, many studies have emerged that either theorize the mathematical aspect of the law or seek new applications for it (e.g., size of 335 rivers, molecular weights of several thousand chemical compounds, or the first digits of the street addresses for the first 342 persons listed in *American Men of Science)*^2^,^3^. The distributions obtained from these datasets were remarkably similar to the predicted frequencies in (1), and those frequencies came to be known as Benford’s Law.

There is ongoing debate about the fundamental origins of BL, but it is clear that it can only be applied to data that fall somewhere between being entirely random (e.g., lottery results) and overly constrained (e.g., the size of new born babies). For many years, little was known about Benford’s Law and its unusual and empirical applications were seen more as a numerical curiosity rather than useful information. In fact, Benford himself called his research paper “The Law of Anomalous Numbers.” More recently it has been established that for real valued continuous data-sets Benford’s law arises naturally if the data are distributed according to a log-uniform modulo 10 distribution^4^.

Based on simulation evidence and measured datasets, studies showed that large classes of naturally occurring quantities (preferentially log-uniform distributed data) are expected to conform to BL^5^. It has been used in forensic accounting for fraud detection or for change detection in physical and natural science phenomena^6^,^7^,^8^,^9^. Several articles have summarized most of the known datasets that follow BL prediction, including river length, population distribution, atomic weight, x-ray volts, American League baseball statistics, black-body radiation, the mass of exoplanets, postal codes, and death rates^5^,^6^,^7^,^8^,^9^. Large datasets of variables that span many orders of magnitude are often seen to follow the distribution^3^,^7^,^10^.

In this study, we test the validity of BL (1) on a natural climatic process. For this purpose we have chosen the traveled distance of tropical cyclones (TC) (Fig. 1), a large dataset available freely online via the International Best Track Archive for Climate Stewardship (IBTrACS).

Describing and understanding a climatic and natural hazard such as tropical cyclone occurrence is a complex task that often requires elaborate mathematical models, especially because of the multifactorial input and intrinsic heterogeneity of the data^11^,^12^,^13^,^14^,^15^. Thus, the quality and homogeneity of the dataset continues to spark heated debates and is frequently used as evidence against newly proposed models. Given the abundance of information that can be extracted from the TC dataset, it is necessary to enhance our ability to understand and separate natural trends from effects due to incomplete and heterogeneous records^11^,^14^. Therefore, in this study we investigate distribution anomalies from Benford’s Law in order to detect temporal changes in the record and monitor the heterogeneity of the TC global dataset.

## Results

**Figure 1:**
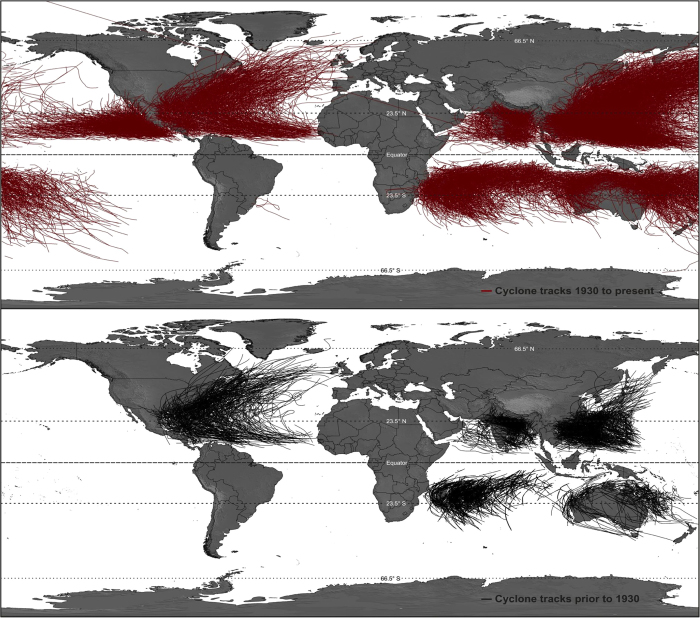
Map of the world TC tracks from International Best Track Archive for Climate Stewardship (IBTrACS). (top) from 1931 to present days; (bottom) from 1841 to 1930; TC records prior to 1931 were based on only a single position estimate per day, while at the same time many parts of the globe were poorly sampled (Jarvinen *et al.*, 1984). There are no data available prior to 1930 for the western Pacific Ocean along the North and Central American coast, while this region is prone to TC activities, especially due to the direct influence of El Niño/La Niña; (Figure made with ArcGIS^®^ software and Corel Draw X5).

**Figure 2:**
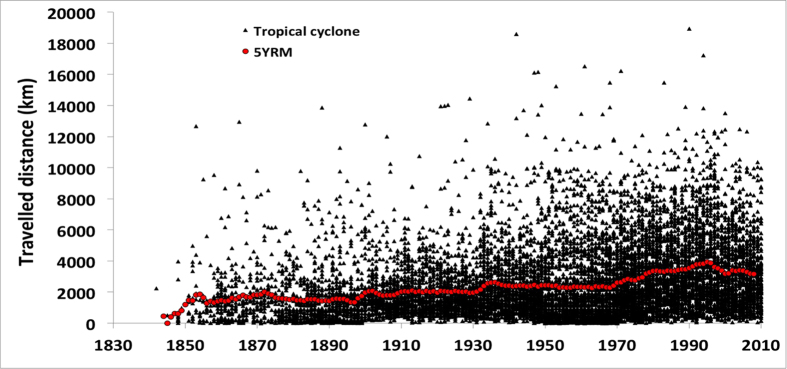
Distribution of TC travelled distances and frequency since 1842 to present. The 1930’s represent a significant improvement in the recording and measurements of tropical cyclone occurrences. Tropical cyclone travelled distances (km) from 1841 to 2010; red curve is the 5 years running mean distance (km).

The distance traveled by each TC was plotted against the year of occurrence (Fig. 2). The number of events increases with time, most likely due to improvements in scientific communications and observational capabilities. For example, only one cyclone track appears in the dataset for 1842 compared to 92 in 1900 and 297 in 1970. We also note that no TC tracks were reported along the Western Pacific coast in the early records (Figs 1 and 2). The minimum and maximum distances traveled in the dataset are 1.2 km and 18,947 km, respectively, spanning over four orders of magnitude. The average distance traveled by cyclones over the complete dataset is 2,560 km but changes from 1,796 km to 2,866 km prior to and after 1931, respectively.

**Figure 3:**
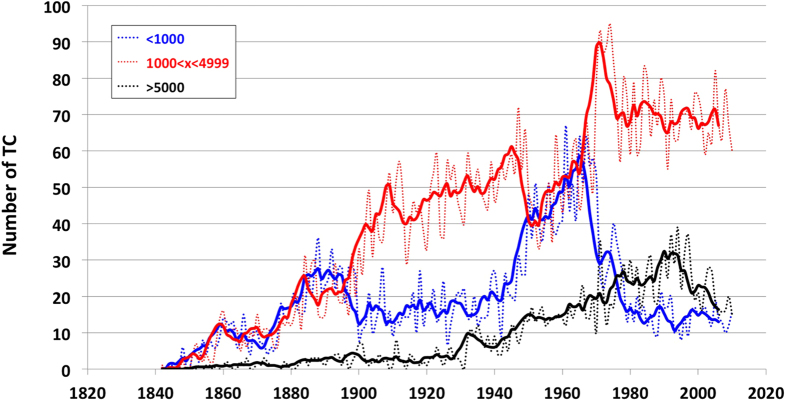
Temporal variations of categories distance travelled by TC. Frequency of TC occurrences relative to the category of distance traveled: (i) short (<1000 kms), (ii) medium (1000 kms < × < 5000 kms) and (iii) long (>5000 kms) (plain curve correspond to the 5 year running mean (5YRM)).

In Fig. 3 we have plotted the evolution of TC tracks over time in three categories of distances traveled: (i) short (<1,000 km), (ii) medium (1,000 km < × < 5,000 km), and (iii) long (>5,000 km). Over time, there has been a change in traveled distances, with a continuous increase in large distances traveled by TCs between 1930 and 2010. Most importantly, a severe and sudden shift occurred in the 1970s between short and medium distances. The overall shift after 1970 is also visible in Fig. 2 as a clear increase in the average distance traveled.

**Figure 4:**
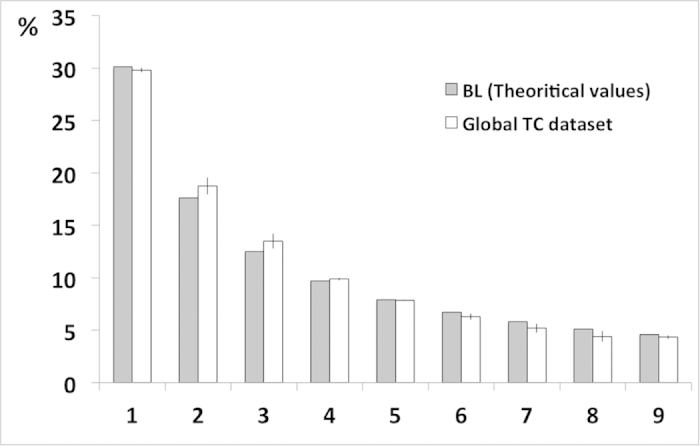
Relationship between BL first digit theoretical values and the first digit TC track distribution. Comparison of the distribution of first digit occurrence in the global TC record with the theoretical Benford’s Law estimates.

Following the calculation of the first-digit occurrence in the TC records, we have compared the distribution with the theoretical values of BL (Fig. 4). One has to keep in mind that BL is scale invariant and that the comparison would not differ in feet, kilometers, or miles. The values in Fig. 4 reveal very little deviation from the theoretical values. In fact, they are in exceptional agreement (for other comparisons see^9^,^16^,^17^), with minimum and maximum absolute differences from empirical values of 0 and 1.2%, respectively, and with an average of 0.51% (see Table 1).

The typical decay of first-digit occurrence can be observed for the complete TC best track data, establishing BL for the TC dataset. This allows the investigation of temporal variation such as potential change-points in the system at specific periods. Similar to the work of Diekmann^18^ as well as Judge and Schechter^19^, we used BL to describe the homogeneity and integrity of the dataset. Once established for a particular dataset, temporary deviations from the theoretical values can potentially indicate additional control processes in the system. The more sudden and stronger the modifications to the system, the more intense and abrupt the BL misfit distribution will vary. For example, the magnitude of, and timing between, earthquakes is in agreement with BL estimates, whereas human activities such as nuclear tests of constant magnitude lead to deviations from the “natural” pattern^7^.

**Figure 5:**
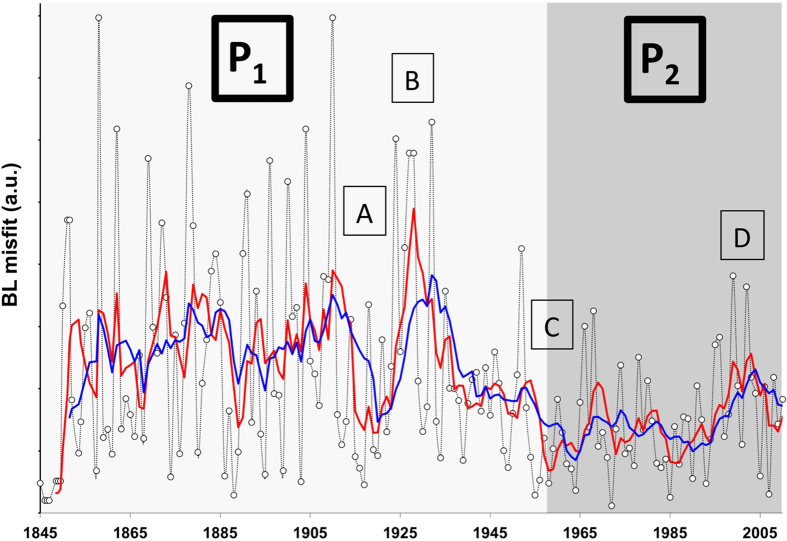
Calculation of misfit from BL estimate over the entire TC dataset. The scatter of the data is on itself an indication of the dataset quality and homogeneity. The graphic represent the evaluation of the BL Misfit per individual years (black with empty circles), 5YRM (red) and 10YRM (blue). **P**_**1**_ and **P**_**2**_ divide the dataset into two large periods relating to technological improvements. A, B, C and D correspond notable shifts or fluctuations within the BL misfit.

Temporal variations in BL estimates between 1842 and 2010 were determined for high-resolution observations by plotting the least square misfit between observed and theoretical first digit distributions over a 5-year and 10-year running window (Fig. 5). This allows the observation of potential episodes within the record that differ from the predicted BL distribution. Those fluctuations are linked to system’s dynamic variations (e.g. incomplete data, change in recording, protocols, unusual activities…) that could go undetected if the dataset is observed in full (see Figs 2 and 3).

## Discussion

Between 1842 and 2010 two distinctive periods, P_1_ (1842-1960) and P_2_ (1960-2010) can be identified (Fig. 5). The data were smoothed over a 5- to 10-year running mean, which makes the period boundaries relatively vague. It is not surprising that the largest deviation from expected first digit distribution is observed in the early record. Not only because the record is incomplete (most TC that did not achieve landfall usually went undetected prior to satellite observation), but also because the TC tracks were built from ship records, sediment archives, and/or observed landfall damages, obviously inducing a large error in the reported distances and trajectories. Landsea and colleagues estimated an undercount bias of 0–5 TCs/yr during 1851–1910 and 0–2 TCs/yr during 1910–1960 by taking into account information on the coastline, TCs, and ship density in the Atlantic basin^20^.

Within the two phases, it seems that short- and long-term trends have occurred at different periods, especially in the first 30 to 40 years of the twentieth century. Four specific episodes can be observed from A to D at 1915, 1925, 1955, and 2000, (+/−5 years) respectively. Episode A in 1915, which spans over a decade, most likely relates to the constant improvement in data coordination and the record of the events, for example, with the increased use of telegraph lines in the early 1900s^20^. Nevertheless, a sudden increase around the mid-1920s (episode B), which represents the most striking feature, cannot be directly explained by data recording or technological improvement. This indicates that the BL misfit could potentially reflect climatic variation within the record. For example, the misfit could be related to a sudden and strong inversion in the El Niño Southern Oscillation (ENSO) record around the mid-1920s^21^. Unfortunately, the comparison of the BL misfit with the climatic record is not sufficient in proving a causal relationship, especially when considering the scatter of the data. Furthermore the agreement between the BL misfit and ENSO record could be a mere coincidence and any attempted interpretation is at best speculation.

Following the sudden increase of BL misfits during episode B in the late 1920s, BL misfits decrease until the 1960s (episode C). This period coincides with the introduction of the aircraft (1944)^22^, especially in the Atlantic basin, and the use of radiosondes in the late 1930s to early 1940s. One has to keep in mind that this was a critical time in history with WWII followed by the Cold War, which led to; (i) technological developments being used for TC detection, such as the radar in mid-1950s; (ii) increased military movement and presence, and therefore a better observation of TC events; and (iii) an improved centralization of the data (i.e. development of computer and military networks, precursors of the internet). With all the technological development it is not surprising to see the BL misfit values drastically and consistently decreasing over time, as the overall quality (e.g. homogeneity, precision, completeness) of the dataset improves.

The two periods pre- and post-1960 can be clearly separated, as misfit values after the mid-1960s are much smaller than those seen in the early record. This change is clearly present in the 1930s and was reinforced in the 1940s. But a divide undoubtedly occurs in the mid-60s, with the smallest BL misfits in the records (apart from 1842, which had only one TC track) being observed. This very abrupt change seen in the mid-1960s is related to profound deviations in the recording system and is indicative of serious effects on the homogeneity, quality, and precision of the TC record. Thus, the period matches the introduction of satellite technology described by Landsea and colleagues^23^,^24^. Measurements of greater precision had a clear impact on the recording of a TC’s distance traveled; it is likely that the early detection of the phenomena increased the overall distance traveled (Fig. 2). It was held that new technologies would contribute to keeping the BL misfit to its lowest values; however Fig. 5 shows the opposite. One potential reason could be that satellite not only offered more detection but also a more precise addition of a clustering of events into extra tropical and subtropical cyclone categories^25^. This would have a direct impact on the number of TCs in the record.

In the late 1990s (episode D, Fig. 5), the BL misfit again peaks to values similar to those observed prior to the introduction of satellites. The advent of new technology can be correlated to the sudden changes, including the deployment of mobile platforms, flight-level instrumentation, Doppler radar systems, aerosondes, and microwave imagery, which have reduced the error of TC tracks and parameter measurements up to 30% in the last two decades^26^,^27^,^28^. However, it appears that not all of the variations observed within the BL misfit relate to technological improvements, but also to the definition of TCs and their data handling. For example, there has been a doubling in the number of TCs in the Atlantic basin over the last century. This increase in the storm count from the original Atlantic basin data has been shown to be mainly due to an increase in short duration (<2-day: “shorties”) tropical storms^29^, which has been attributed to changes in observation capabilities^30^. The appearance of “shorties” in the TC data record have become even more pronounced in the last decades due to the aforementioned technological improvements. The impact of this artificial increase in short-lived Atlantic basin TCs reduces the mean track length, which is seen in the decrease from 1995 onward (Figs 2 and 3) and leads to strong variation in the BL misfit (Fig. 5, BL variations are increasing steadily until peaking around 2000 +/−5 yr). Interestingly, this strong variation also coincides with the “super El Nino” episodes of 1998 that started in the early 1990s. Again, the BL misfit peaks or trend could be influenced by both technological improvements and climatic variations, however, it is difficult to tease them apart.

It is most likely that technological improvement and climatic variation will have different impacts that yield significant variation within the pattern of BL misfits. Human-induced climatic variations potentially provoke a deviation significantly different from what we could name “natural” variation. For instance, the fact that improvements in measurement methods and technology happen very suddenly could influence BL misfits differently than a gradual anthropogenic influence on the climate. A non-uniform variation (e.g., the uninterrupted increase of medium and long distance TCs while short distance TCs have for the most part remained constant Fig. 3) could be due to changes in climatic processes, such as an increases in sea surface temperature (SST) or prolonged atmospheric pressure anomalies. Although, one could easily argue the opposite, that recording precision could obviously be responsible for such a change. In this study, the TC record appears too complex and heterogeneous to be corrected by directly using the BL misfit. Nevertheless, BL offers an innovative approach to assess the TC dataset, and clearly helps to identify the influence of technological improvement in measurement capabilities. Furthermore, using BL misfits we can quantify which technology had the most influence on dataset integrity. According to Fig. 5, satellite introduction was clearly the most remarkable change in the TC record.

Given that climatic indicators are likely to be reflected in the TC record, it appears that the key limitation lies in our ability to understand and extract climatically influenced data from the record. A mathematical system such as BL offers a new approach to assess errors and discrepancies. Most of all, it enables us to map variations in the record not observable by classical statistical approaches, allowing us to define the most suited part of the dataset for detailed studies. In some circumstances, BL could offer the ability to identify specific periods of biased records that need to be excluded or corrected in order to accurately model the dataset. For example, BL shows that the early part of the record (before 1915) should be use with extreme caution in climatic models and TC statistics. The early records are known to be heterogeneous, and are obviously incomplete due to limited measurement capabilities, while 1915 onwards offers a much more stable part of the TC record. The ability to identify parts of biased data within the records (and perhaps to correct for it) using BL could enhance climatic modelling capabilities to extract crucial information about TC occurrences. Examining datasets for instrumentation artifacts, especially in the case of TCs, could potentially allow us to isolate climatic influences such as ENSO variation, anthropogenic activities, and other climatic variation more accurately.

## Conclusion

Benford’s Law presents a new way to investigate and assess the homogeneity and quality of natural hazard datasets. While we have shown that BL prediction over the complete dataset is verifiable, a large deviation within the temporal record can clearly be attributed to technological improvements. The introduction of new instrumentation, such as satellite observations, has had a large impact on the dataset quality and is clearly reflected in the form of strong variations in the BL misfit. Furthermore, the quality of discrete timespans within the dataset can be evaluated using BL, as demonstrated by the fact that the clustering of TC events resulting from measurement precision was also clearly observable in the BL misfit. To conclude, we can say that the use of mathematical laws, such as Benford’s Law, has the potential to identify changes in natural systems and could possibly offer the ability to correct for a heterogeneous dataset. Thus BL can be used to select the appropriate part of a large dataset to run climatic models or to account for the impact of a known transition in the system. While strong natural climatic variation and anthropogenic impact on TC occurrences was not clearly observable at this point in the BL misfit, long deviation trends, and/or sudden peaks could potentially be linked to climatic processes in the future.

This type of analysis enables one to observe temporal or spatial variations in large data sets with sufficient dynamic range. However, it does not have any predictive power, and does not tell us anything about future climate change. It is a tool for detection, rather than prediction. Benford’s law has already been exploited to detect signals hidden in background noise in other time series data. For example, Sambridge and colleagues^7^ showed how seismic energy from an earthquake can be detected from just the first digit distribution of displacement counts on a seismometer. We therefore expect this approach to be a powerful tool used to detect unknown anomalies or abrupt changes in climate data, and we anticipate new applications to appear in this ever-growing field of research.

## Methods

### TC Records

Global TC tracking information from 1842 to 2010 was obtained from the International Best Track Archive for Climate Stewardship. The geometric path of each event can be downloaded from the IBTrACS website, resulting in a dataset consisting of more than 350,000 data points that describe the geometry of each path (Fig. 1). Using the averaged radius for the Earth of 6,371 km, we have calculated the distance along the great circle between each consecutive point and have computed the total distance traveled by cyclones. TCs defined by only one point were excluded from the calculation in order to avoid introducing artificial values that would offset the first-digit count. The total number of independent occurrences available to us was n = 11,863 at the time of the study.

### BL Calculation

Following BL, the distribution of first-digit values is defined by the probability function (1), where *b* is the base (here 10) and *d* the leading digit. The theoretical distribution gives a frequency of occurrence of 30.1% for digit 1, 17.6% for 2, 12.5% for 3, and so on, until reaching 4.6% for the ninth digit. This law is scale and base invariant; thus it is independent of the units used (e.g., miles or kilometers).

A least-squares misfit measure (2) is used to quantify the discrepancy between the observed and predicted first-digit proportions:
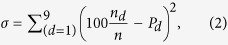
where *P*_*d*_ is the expected proportion of data with first digit *d* as given by BL theoretical values, *n*_*d*_ is the number of observed data with first digit *d*, and *n* is the total number of data. We acknowledge that this measure of goodness-of-fit is arbitrary. Here it is solely intended to quantify the relative distance to Benford’s law between different subsets of data. Little is known about data uncertainties, and hence this quantity cannot be used to measure an absolute goodness-of-fit, through for example a statistical significance test (e.g. chi-square test).

## Additional Information

**How to cite this article**: Joannes-Boyau, R. *et al*. Using Benford's law to investigate Natural Hazard dataset homogeneity. *Sci. Rep*. **5**, 12046; doi: 10.1038/srep12046 (2015).

## Figures and Tables

**Table 1:** First digit values obtained over the entire TC dataset (Empirical values) against the theoretical values describe by Benford’s law.

First digit	Theoretical values %	Empirical values %
1	30.1	29.8
2	17.6	18.8
3	12.5	13.5
4	9.7	9.8
5	7.9	7.9
6	6.7	6.3
7	5.8	5.2
8	5.1	4.4
9	4.6	4.3
